# Estimating the burden of influenza‐associated hospitalizations and deaths in Central America

**DOI:** 10.1111/irv.12385

**Published:** 2016-03-29

**Authors:** Miguel A. Descalzo, Wilfrido Clara, Guiselle Guzmán, Ricardo Mena, Julio Armero, Bredy Lara, Carlos Saenz, Anabela Aragón, Rafael Chacón, Nathalie El‐Omeiri, Jairo Méndez‐Rico, Mauricio Cerpa, Rakhee Palekar, Jorge Jara, Eduardo Azziz‐Baumgartner

**Affiliations:** ^1^ Universidad del Valle de Guatemala (UVG)Guatemala CiudadGuatemala; ^2^ U.S. CDC Regional Office for Central America Region (CDC‐CAR)Guatemala CiudadGuatemala; ^3^ Caja Costarricense del Seguro Social (CCSS)San JoséCosta Rica; ^4^ Ministerio de Salud Pública y Asistencia Social de Guatemala (MSPAS)Guatemala CiudadGuatemala; ^5^ Ministerio de Salud de El Salvador (MINSAL)San SalvadorEl Salvador; ^6^ Secretaría de Salud de HondurasTegucigalpaHonduras; ^7^ Ministerio de Salud de Nicaragua (MINSA)ManaguaNicaragua; ^8^ Pan American Health OrganizationWashingtonDCUSA; ^9^ U.S. Centers for Disease Control and Prevention (CDC)AtlantaGAUSA

**Keywords:** America, burden of disease, hospitalizations, influenza, meta‐analysis, mortality, multiplier model

## Abstract

**Objectives:**

Our objective was to estimate the incidence of influenza‐associated hospitalizations and in‐hospital deaths in Central American Region.

**Design and setting:**

We used hospital discharge records, influenza surveillance virology data, and population projections collected from Costa Rica, El Salvador, Guatemala, Honduras, and Nicaragua to estimate influenza‐associated hospitalizations and in‐hospital deaths. We performed a meta‐analysis of influenza‐associated hospitalizations and in‐hospital deaths.

**Main outcome measures:**

The highest annual incidence was observed among children aged <5 years (136 influenza‐associated hospitalizations per 100 000 persons).

**Results:**

Annually, 7 625–11 289 influenza‐associated hospitalizations and 352–594 deaths occurred in the subregion.

**Conclusions:**

Our results suggest that a substantive number of persons are annually hospitalized because of influenza. Health officials should estimate how many illnesses could be averted through increased influenza vaccination.

## Introduction

Influenza is a vaccine preventable disease that annually causes approximately 1 million influenza‐associated hospitalizations among children younger than 5 years and 500 000 deaths among all age‐groups.^1^, ^2^ The burden of disease is concentrated predominantly in developing countries where an estimated 934 000 hospitalizations and 111 500 deaths attributable to influenza occur among children aged <5 years.^1^

Few studies have estimated the burden of influenza illness in Central America, a tropical region comprised of lower to upper‐middle‐income countries.^3^, ^4^, ^5^, ^6^ Estimating the influenza disease burden is difficult because ill persons may not seek care or present for care with ill‐defined symptoms where they are often treated empirically without respiratory sampling. Information on disease burden can be useful for local public health practitioners exploring the potential value of prevention programs.^6^, ^7^

Annual influenza vaccination remains a key strategy for the prevention of influenza‐related hospitalizations and deaths. In addition since 2004, countries in the Americas have expanded the use of influenza vaccine among subpopulations at increased risk of complications following influenza infections.^8^ As annual influenza vaccination is a costly investment, influenza disease burden should be well documented,^9^ as should be the assessment of influenza vaccine impact.^7^

Our objective was to estimate the incidence of influenza‐associated hospitalizations and in‐hospital deaths in five Central American countries.

## Methods

We used hospital discharge records, influenza surveillance virology data and population projections collected during January 1, 2009, to December 31, 2012, from Costa Rica, El Salvador, Guatemala, Honduras, and Nicaragua to estimate influenza‐associated hospitalizations and in‐hospital deaths.^6^

### Calculating the number of pneumonia and influenza diagnoses

In all countries except Guatemala, we obtained public hospital discharge records and in‐patient survival data from national Health Information Systems available from the Ministries of Health. In Guatemala, we recruited a survey team to collect the information at all public hospitals. For each month during 2009–2012, we determined the number of patients hospitalized with pneumonia or influenza diagnoses corresponding to ICD‐10 codes J09–J18, the WHO proxies for severe acute respiratory infections (SARI),^6^ by age‐group, that is, <5 years, 5–64 years, and >64 years, the age‐groups typically targeted for vaccination.^8^

### Estimating the proportion of hospitalized patients with influenza virus infection using surveillance data

Influenza surveillance among SARI case‐patients was conducted in the study countries by sentinel hospitals and National Influenza Centers according to PAHO‐CDC 2006 generic protocol for influenza surveillance.^3^, ^6^ Laboratories tested respiratory samples using immunofluorescence and rtRT‐PCR^3^, ^6^ after systematically identifying a sample of SARI cases through routine clinical practice. We determined the age‐stratified percentage of samples from SARI case‐patients testing positive for influenza virus by dividing the number of positive by the total sampled each same month.

### Calculating the population at risk based upon population projections

We assumed that every inhabitant was at risk of developing severe influenza illness and requiring admission to a public hospital. Thus, we defined the population at risk per country as the most recent population estimate based on census projections, stratified by year and age‐group. National Statistical Offices provided the data for each country. As a sensitivity analysis, we also assumed that in each country, only the proportion of the population typically seeking care at public hospitals network was at risk of developing severe influenza and being identified in national data sets. Public hospitals cover 87% of the population in Costa Rica, 80% in El Salvador, 70% in Guatemala, 60% in Honduras, and 61% in Nicaragua.^10^

### Statistical analysis

We estimated incidence of influenza‐associated hospitalizations and in‐hospital deaths for each year, age‐group, and country by multiplying the age‐stratified monthly number of patients with pneumonia or influenza clinical diagnoses by the age‐stratified percentage of samples from SARI surveillance case‐patients that tested positive for influenza. We then divided the estimated annual number of influenza‐associated hospitalizations and deaths by the population at risk. The formula is presented in the Data S1. We calculated 95% confidence intervals (95% CI) assuming a Poisson distribution. We performed a meta‐analysis of individual country rates by pooling the information (2009–2012) from the five countries, using a random‐effects model, and assuming significant heterogeneity in the data. We conducted a second sensitivity analysis stratifying influenza rates into pandemic (2009–2010) and seasonal influenza years (2011–2012).

### Ethical issues

The project was reviewed by Institutional Review Committees of the Universidad del Valle de Guatemala and the countries' Ministries of Health and judged to be a public health evaluation. Only records without personal identifiers were used for the analysis.

## Results

### Pneumonia and influenza diagnoses

During 2012, Costa Rica, El Salvador, Guatemala, Honduras, and Nicaragua had 40 325 186 inhabitants: 4 951 130 (12%) children aged <5 years, 33 299 080 (83%) persons aged 5–64 years, and 2 074 976 (5%) adults aged >64 years. During 2009–2012, there were 313 453 hospitalizations with pneumonia and influenza diagnoses, of which 13 218 (4%) resulted in death during hospitalization (Tables S1–S5).

Children aged <5 years comprised the greatest proportion (226 026 [72%]) of the 313 453 pneumonia and influenza hospitalizations. Older adults aged >64 years comprised the greatest proportion (5532 [42%]) of the 13 218 in‐hospital deaths, followed by children aged <5 years (4092 [31%]). The age‐stratified percentage of hospitalizations and in‐hospital deaths varied significantly between countries (Data S1) (*P*‐value<0·001).

### SARI case‐patients under surveillance

**Figure 1:**
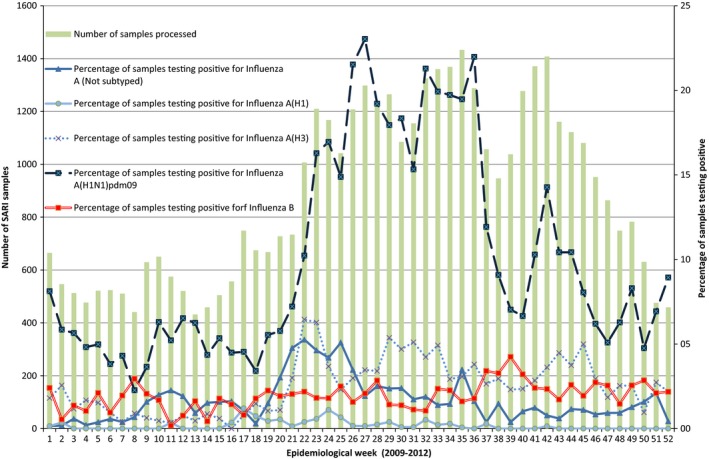
Distribution of samples testing positive for influenza by type/subtype in Central America. Bars represent the number of SARI cases samples processed in surveillance. Lines represent the percentage of samples testing positive for influenza. Each line represents a different type/subtype of virus.

During the study period, surveillance identified and tested 43 951 SARI case‐patients samples for influenza; 48% children aged <5 years, 41% persons aged 5–64 years, and 11% adults aged >64 years. The percentages of SARI case‐patients that tested positive for influenza viruses varied significantly between age‐groups (*P*‐value<0·001): 9% among children aged <5 years, 32% among persons aged 5–64 years, and 13% among adults aged >64 years. In 2009 and 2011 influenza A (H1N1) pdm09 was the most commonly identified subtype among SARI case‐patients samples (Figure 1). In 2010, influenza A (H3) was detected in 6% and in 2012 influenza B appeared in 4% of all samples processed.

### Influenza‐associated hospitalizations

**Figure 2:**
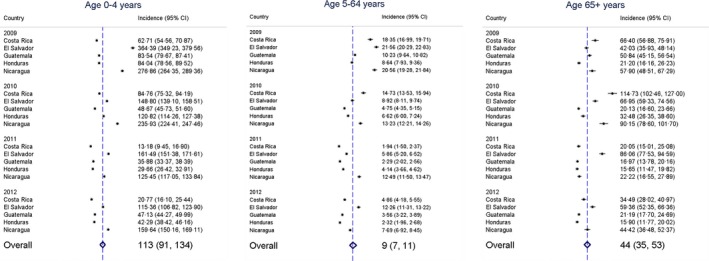
Incidence of influenza‐associated hospitalizations by age‐groups in five Central American countries during 2009–2012. Dots represent estimates of influenza‐associated hospitalizations by country and year per 100 000 persons. Diamonds represent overall meta‐analysis estimate of influenza‐associated hospitalizations using random‐effects analysis.

Annually approximately 9505 persons (95% CI: 7563–11 397) were hospitalized in the five countries with influenza‐associated SARI. Among them, 5595 (59%) were children aged <5 years, 2997 (31%) persons aged 5–64 years, and 913 (10%) adults aged >64 years. The highest incidence of influenza‐associated hospitalizations occurred among children aged <5 years (113 [95% CI: 91–134] per 100 000 children) which was 12‐fold higher than among persons aged 5–64 years (*P* < 0·001) (Figure 2).

### Influenza‐associated deaths

**Figure 3:**
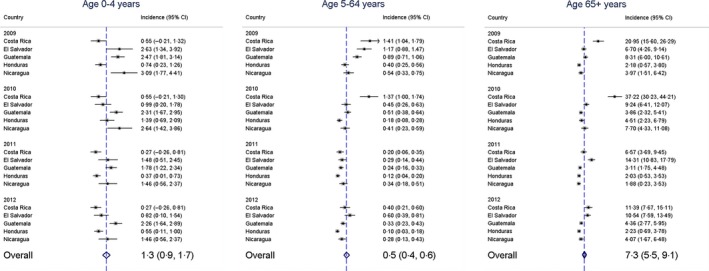
Incidence of influenza‐associated deaths by age‐groups in five Central American countries during 2009–2012. Dots represent estimates of influenza‐associated deaths by country and year per 100 000 persons. Diamonds represent overall meta‐estimate of influenza‐associated deaths using random‐effects analysis.

Annually approximately 382 persons (95% CI: 292–473) died during their influenza‐associated hospitalization in the five countries. A total of 64 (17%) in‐hospital deaths occurred among children aged <5 years, 166 (43%) among persons aged 5–64 years, and 151 (40%) among adults aged >64 years. The highest influenza‐associated mortality occurred among adults aged >64 years (7·3 [95% CI: 5·5–9·1] per 100 000 persons) and was 14‐fold higher than among persons aged 5–64 years (*P* < 0·001) (Figure 3).

Influenza‐associated hospitalization and in‐hospital deaths rates were 1·4–1·5 higher if we assumed only the subpopulation seeking care at public hospitals was at risk of developing illness identifiable through our national data. (Table S6).

## Discussion

Our results suggest that influenza, a vaccine preventable illness, is associated with substantial morbidity and mortality in Central America. In the five countries studied, 7563–11 397 influenza‐associated hospitalizations and 292–473 in‐hospital deaths occurred annually among 40 million inhabitants.

Very young children were most commonly hospitalized as a result of influenza‐associated hospitalizations and older adults most commonly died as a result of influenza. While persons aged 5–64 years were more commonly hospitalized during 2009 because of influenza A (H1N1) pdm09 influenza pandemic,^11^ we did not identify a difference in the annual age‐adjusted influenza‐associated hospitalization rates by influenza type and subtype during sensitivity analyses (Table S6).

Influenza‐associated hospitalizations rates in this study were similar to those published in a recent global meta‐analysis^1^ which reported a rate of 1 (95% CI: 1–2) per 1000 person‐years among children aged <5 years. Costa Rica used similar methods^6^ and obtained comparable results. El Salvador and Nicaragua^3^, ^4^ estimated slightly higher rates than our results, whereas another study conducted in Guatemala^5^ estimated slightly lower rates. These differences could be explained by the use of active population‐based surveillance in these studies which may have resulted in increased referrals of case‐patients to the hospital.

Our estimated influenza‐associated in‐hospital mortality was similar to that reported previously in the region, except for the lower mortality rates we estimated among adults aged >64 years.^6^, ^12^, ^13^ Our rates among children aged <5 years were also lower than those of a global meta‐analysis using different methodology,^1^ whereby 4·9 children per 100 000 died from influenza‐associated severe acute lower respiratory infection (ALRI) in developing countries. A plausible reason for these discrepancies could be the incompleteness and underreporting in mortality registries in study countries.^14^ In addition, ALRI tends to be more sensitive than SARI in identifying influenza illness and tends to yield higher rate estimates.

### Limitations

We extrapolated influenza laboratory results from SARI case‐patients identified through surveillance to all untested hospitalized patients with SARI proxy discharge diagnoses. We were unable to assess differences between SARI case‐patients identified through surveillance and the SARI proxies. In addition, surveillance systems and medical practices vary from country to country; we therefore used random‐effects models because we assumed that influenza‐associated hospitalization rates incidence varied by country but followed same distribution. Our results might underestimate the burden of influenza‐associated hospitalizations and mortality because underreporting of deaths is common in developing countries; a study estimated ~50% underreporting in 2003 for some countries in the region.^14^ Moreover, we assessed only in‐hospital deaths and missed those that occurred in the community. Last approximately 20% of samples were analyzed using indirect immunofluorescence, an assay that has a lower sensitivity than rtRT‐PCR.^15^

## Conclusion

Influenza is among the leading causes of hospitalizations and deaths.^2^ Our results suggest that, each year, a substantial number of persons in Central America are hospitalized and died as a result of influenza. Our findings suggest that public health professionals should evaluate the potential value of giving young children and older adults influenza vaccines and empiric treatment with antivirals. Further studies should be conducted to explore the number of illnesses and cost averted through influenza vaccination and antiviral policies especially among subpopulations at high risk of developing complications as a result of influenza illness (e.g., pregnant women).

## Funding

This work was supported by cooperative agreement between Centers for Disease Control and Prevention and Universidad del Valle de Guatemala “Strengthening Infectious Disease Research Capacity for Public Health in Guatemala and Central America” No. 1U01GH000028‐04.

## Authors' contributions

MAD had full access to all the data in the study and takes responsibility for the integrity of the data and the accuracy of the data analysis. MAD, WC, JJ, and EAB contributed to study concept and design. GG, RM, JA, BL, CS, AA, RC, MC, and JM contributed to acquisition of data. MAD, WC, GG, RM, JA, BL, CS, AA, RC, NEO, JM, MC, RP, JJ, and EAB contributed to analysis and interpretation of data. MAD drafted the manuscript. MAD, WC, GG, RM, JA, BL, CS, AA, RC, NEO, JM, MC, RP, JJ, and EAB critically revised the manuscript for important intellectual content. MAD performed statistical analysis. MAD, WC, and JJ contributed to study supervision.

## Conflicts of interest

None of the authors have any conflicting interests to declare.

## Supporting information

Data S1. Mathematical formula
**Table S1.** Influenza‐associated hospitalizations and deaths in Costa Rica during 2009–2012.
**Table S2.** Influenza‐associated hospitalizations and deaths in El Salvador during 2009–2012.
**Table S3.** Influenza‐associated hospitalizations and deaths in Guatemala during 2009–2012.
**Table S4.** Influenza‐associated hospitalizations and deaths in Honduras during 2009–2012.
**Table S5.** Influenza‐associated hospitalizations and deaths in Nicaragua during 2009–2012.
**Table S6.** Sensitivity analysis of influenza‐associated hospitalizations and deaths.
