# Supplementary material for: Estimating the burden of influenza‐associated hospitalizations and deaths in Central America
Source: Influenza Other Respir Viruses. 2016 Mar 29;10(4):340–5. doi: 10.1111/irv.12385 (PMC4910178; doi:10.1111/irv.12385)
Supplement: Supplementary file 1 — Data S1. Mathematical formula Table S1. Influenza‐associated hospitalizations and deaths in Costa Rica during 2009–2012. Table S2. Influenza‐associated hospitalizations and deaths in El Salvador during 2009–2012. Table S3. Influenza‐associated hospitalizations and deaths in Guatemala during 2009–2012. Table S4. Influenza‐associated hospitalizations and deaths in Honduras during 2009–2012. Table S5. Influenza‐associated hospitalizations and deaths in Nicaragua during 2009–2012. Table S6. Sensitivity analysis of influenza‐associated hospitalizations and deaths. [file IRV-10-340-s001.docx]

**Supplementary material**

Once we collected all information from the three secondary data sources, incidence was calculated in every country using the following formula:

$$I_{a}= \frac{\sum_{m} N_{a,m}*\frac{P_{a,m}}{T_{a,m}}}{C_{a}}$$

Where

I_a_ incidence of influenza-associated hospitalizations,

N_a_,_m_ number of hospital discharge records (hospitalizations) with a diagnosis of SARI proxy (J09-J18 codes from ICD-10) by age group and month,

P_a,m_ number of tested samples which were positive for influenza virus by age group and month,

T_a,m_ number of tested SARI case-patients samples by age group and month,

C_a_ population projections,

a age group (< 5 years old, 5-64 and >64 years),

m month

The same formula was used to estimate influenza-associated in-hospital mortality, but in this case, N_a,m_ was the number of in-hospital deaths with a diagnosis of SARI proxy (J09-J18 codes from ICD-10).

**Table 1: Influenza-associated hospitalizations and deaths in Costa Rica during 2009–2012**

| Year | Age group (years) | Population size ^a^ | Severe acute respiratory infection | | Annual percentage of influenza positive respiratory samples ^d^ | Influenza-associated hospitalizations ^e^ | | Influenza-associated deaths ^f^ | |
| --- | --- | --- | --- | --- | --- | --- | --- | --- | --- |
|  |  |  | Hospitalizations ^b^ | Mortality ^c^ |  | Number  (95% CI) | Rate (95% CI) | Number  (95% CI) | Rate (95% CI) |
|  | <5 | 361963 | 1770 | 9 | 191/1341 (14) | 227 (198 - 257) | 62.8 (54.6 - 70.9) | 2 (-1 - 4) | 0.4 (-0.2 - 1.1) |
| 2009 | 5-64 | 3825728 | 1963 | 150 | 1632/3759 (43) | 702 (647 - 754) | 18.3 (16.9 - 19.7) | 54 (38 - 65) | 1.4 (1 - 1.7) |
|  | >64 | 281646 | 1485 | 466 | 104/694 (15) | 187 (161 - 214) | 66.5 (57 - 76) | 59 (43 - 74) | 20.8 (15.4 - 26.1) |
|  | <5 | 365751 | 2139 | 12 | 187/1241 (15) | 310 (275 - 344) | 84.6 (75.2 - 94.1) | 2 (0 - 5) | 0.5 (-0.1 - 1.3) |
| 2010 | 5-64 | 3875276 | 1784 | 166 | 533/1590 (34) | 571 (523 - 616) | 14.7 (13.5 - 15.9) | 53 (39 - 66) | 1.3 (1 - 1.7) |
|  | >64 | 292867 | 1732 | 574 | 84/507 (17) | 336 (300 - 372) | 114.7 (102.5 - 127) | 109 (88 - 129) | 37.1 (30.1 - 44.1) |
|  | <5 | 364292 | 1721 | 14 | 49/1801 (3) | 48 (35 - 62) | 13.2 (9.5 - 17) | 1 (-1 - 2) | 0.1 (-0.2 - 0.5) |
| 2011 | 5-64 | 3923546 | 1369 | 150 | 44/759 (6) | 76 (55 - 90) | 1.9 (1.4 - 2.3) | 8 (0 - 12) | 0.2 (0 - 0.3) |
|  | >64 | 304311 | 1487 | 508 | 19/435 (4) | 61 (46 - 76) | 20.1 (15.1 - 25.1) | 20 (11 - 29) | 6.5 (3.6 - 9.4) |
|  | <5 | 365896 | 1575 | 9 | 79/1526 (5) | 76 (58 - 92) | 20.6 (15.9 - 25.2) | 1 (-1 - 2) | 0.1 (-0.2 - 0.5) |
| 2012 | 5-64 | 3970531 | 1343 | 118 | 170/1139 (15) | 193 (163 - 218) | 4.8 (4.1 - 5.5) | 16 (8 - 20) | 0.3 (0.2 - 0.5) |
|  | >64 | 316031 | 1457 | 522 | 52/723 (7) | 109 (88 - 129) | 34.3 (27.9 - 40.8) | 36 (24 - 48) | 11.3 (7.6 - 15.1) |

^a^ Costa Rica census projections

^b^ Number of persons hospitalized during 2009–2012 with severe acute respiratory infection (SARI) proxy diagnoses (ICD-10 codes J9-18)

^c^ Number of deaths among persons hospitalized during 2009–2012 with SARI proxy diagnoses (ICD-10 codes J9-18)

^d^ Number of nasal and pharyngeal specimens annually positive for influenza through immunofluorescence and polymerase chain reaction over total number tested (percentage)

^e^ Estimated by the product of the proportion of samples testing positive for influenza and the number of persons hospitalized with by age-group and month (95% confidence interval). Rate per 100,000 habitants

^f^ Estimated by the product of the proportion of samples testing positive for influenza and the number of persons dying by age-group and month (95% confidence interval). Rate per 100,000 habitants

**Table 2: Influenza-associated hospitalizations and deaths in El Salvador during 2009–2012**

| Year | Age group (years) | Population size ^a^ | Severe acute respiratory infection | | Annual percentage of influenza positive respiratory samples ^d^ | Influenza-associated hospitalizations ^e^ | | Influenza-associated deaths ^f^ | |
| --- | --- | --- | --- | --- | --- | --- | --- | --- | --- |
|  |  |  | Hospitalizations ^b^ | Mortality ^c^ |  | Number  (95% CI) | Rate (95% CI) | Number  (95% CI) | Rate (95% CI) |
|  | <5 | 608407 | 15730 | 124 | 128/775 (17) | 2217 (2124 - 2308) | 364.3 (349.1 - 379.4) | 16 (8 - 24) | 2.6 (1.3 - 3.9) |
| 2009 | 5-64 | 5111148 | 3167 | 185 | 499/1048 (48) | 1102 (1032 - 1165) | 21.5 (20.2 - 22.8) | 60 (41 - 72) | 1.1 (0.8 - 1.4) |
|  | ≥65 | 432997 | 2040 | 339 | 23/147 (16) | 182 (155 - 208) | 41.9 (35.8 - 48) | 29 (19 - 40) | 6.7 (4.3 - 9.2) |
|  | <5 | 606849 | 10685 | 55 | 82/949 (9) | 903 (844 - 962) | 148.8 (139.1 - 158.5) | 6 (1 - 10) | 0.9 (0.1 - 1.6) |
| 2010 | 5-64 | 5132510 | 2655 | 138 | 116/531 (22) | 458 (416 - 498) | 8.9 (8.1 - 9.7) | 23 (10 - 31) | 0.4 (0.2 - 0.6) |
|  | ≥65 | 443639 | 2393 | 375 | 40/280 (14) | 297 (264 - 331) | 67 (59.4 - 74.6) | 41 (28 - 54) | 9.3 (6.4 - 12.1) |
|  | <5 | 606218 | 14789 | 133 | 73/1017 (7) | 979 (917 - 1040) | 161.4 (151.3 - 171.5) | 9 (2 - 15) | 1.4 (0.4 - 2.4) |
| 2011 | 5-64 | 5155624 | 2397 | 127 | 33/229 (14) | 302 (263 - 335) | 5.8 (5.1 - 6.5) | 15 (5 - 21) | 0.2 (0.1 - 0.4) |
|  | ≥65 | 454312 | 2181 | 363 | 16/104 (15) | 391 (352 - 430) | 86.1 (77.5 - 94.6) | 65 (49 - 80) | 14.2 (10.7 - 17.7) |
|  | <5 | 607671 | 9924 | 75 | 99/1274 (8) | 701 (649 - 752) | 115.3 (106.8 - 123.8) | 5 (0 - 9) | 0.7 (0 - 1.4) |
| 2012 | 5-64 | 5178841 | 2905 | 138 | 172/637 (27) | 635 (585 - 684) | 12.2 (11.3 - 13.2) | 31 (16 - 41) | 0.5 (0.3 - 0.8) |
|  | ≥65 | 464988 | 2150 | 411 | 21/129 (16) | 276 (243 - 309) | 59.3 (52.3 - 66.4) | 49 (35 - 62) | 10.5 (7.5 - 13.4) |

^a^ El Salvador census projections

^b^ Number of persons hospitalized during 2009–2012 with severe acute respiratory infection (SARI) proxy diagnoses (ICD-10 codes J9-18)

^c^ Number of deaths among persons hospitalized during 2009–2012 with SARI proxy diagnoses (ICD-10 codes J9-18)

^d^ Number of nasal and pharyngeal specimens annually positive for influenza through immunofluorescence and polymerase chain reaction over total number tested (percentage)

^e^ Estimated by the product of the proportion of samples testing positive for influenza and the number of persons hospitalized with SARI by age-group and month (95% confidence interval). Rate per 100,000 habitants

^f^ Estimated by the product of the proportion of samples testing positive for influenza and the number of persons dying by age-group and month (95% confidence interval). Rate per 100,000 habitants

**Table 3: Influenza-associated hospitalizations and deaths in Guatemala during 2009–2012**

| Year | Age group (years) | Population size ^a^ | Severe acute respiratory infection | | Annual percentage of influenza positive respiratory samples ^d^ | Influenza-associated hospitalizations ^e^ | | Influenza-associated deaths ^f^ | |
| --- | --- | --- | --- | --- | --- | --- | --- | --- | --- |
|  |  |  | Hospitalizations ^b^ | Mortality ^c^ |  | Number  (95% CI) | Rate (95% CI) | Number  (95% CI) | Rate (95% CI) |
|  | <5 | 2142773 | 17819 | 554 | 121/1245 (10) | 1790 (1706 - 1871) | 83.5 (79.6 - 87.3) | 53 (36 - 66) | 2.4 (1.7 - 3.1) |
| 2009 | 5-64 | 11272475 | 4423 | 393 | 133/516 (26) | 1153 (1082 - 1217) | 10.2 (9.6 - 10.8) | 100 (79 - 113) | 0.8 (0.7 - 1) |
|  | ≥65 | 601849 | 1644 | 238 | 17/78 (22) | 306 (272 - 341) | 50.9 (45.2 - 56.6) | 50 (36 - 64) | 8.3 (6 - 10.6) |
|  | <5 | 2165745 | 15364 | 715 | 122/1675 (7) | 1054 (990 - 1118) | 48.6 (45.7 - 51.6) | 50 (35 - 63) | 2.3 (1.6 - 2.9) |
| 2010 | 5-64 | 11574956 | 4339 | 462 | 63/501 (13) | 550 (498 - 590) | 4.7 (4.3 - 5.1) | 59 (35 - 69) | 0.5 (0.3 - 0.6) |
|  | ≥65 | 620965 | 1655 | 311 | 10/133 (8) | 125 (103 - 147) | 20.1 (16.6 - 23.6) | 24 (14 - 33) | 3.8 (2.2 - 5.3) |
|  | <5 | 2187869 | 15895 | 674 | 55/1155 (5) | 785 (729 - 838) | 35.8 (33.3 - 38.3) | 39 (26 - 50) | 1.7 (1.2 - 2.3) |
| 2011 | 5-64 | 11883590 | 3935 | 415 | 32/442 (7) | 272 (238 - 297) | 2.2 (2 - 2.5) | 29 (12 - 36) | 0.2 (0.1 - 0.3) |
|  | ≥65 | 642304 | 1628 | 297 | 9/165 (5) | 109 (89 - 130) | 17 (13.8 - 20.2) | 20 (11 - 29) | 3.1 (1.7 - 4.5) |
|  | <5 | 2208844 | 12606 | 621 | 75/902 (8) | 1041 (976 - 1104) | 47.1 (44.2 - 50) | 50 (35 - 64) | 2.2 (1.6 - 2.9) |
| 2012 | 5-64 | 12199249 | 3999 | 403 | 62/493 (13) | 434 (390 - 464) | 3.5 (3.2 - 3.8) | 40 (24 - 49) | 0.3 (0.2 - 0.4) |
|  | ≥65 | 665281 | 1602 | 309 | 15/164 (9) | 141 (117 - 164) | 21.1 (17.6 - 24.6) | 29 (19 - 39) | 4.3 (2.8 - 5.9) |

^a^ Guatemala census projections

^b^ Number of persons hospitalized during 2009–2012 with severe acute respiratory infection (SARI) proxy diagnoses (ICD-10 codes J9-18)

^c^ Number of deaths among persons hospitalized during 2009–2012 with SARI proxy diagnoses (ICD-10 codes J9-18)

^d^ Number of nasal and pharyngeal specimens annually positive for influenza through immunofluorescence and polymerase chain reaction over total number tested (percentage)

^e^ Estimated by the product of the proportion of samples testing positive for influenza and the number of persons hospitalized with SARI by age-group and month (95% confidence interval). Rate per 100,000 habitants

^f^ Estimated by the product of the proportion of samples testing positive for influenza and the number of persons dying by age-group and month (95% confidence interval). Rate per 100,000 habitants

**Table 4: Influenza-associated hospitalizations and deaths in Honduras during 2009–2012**

| Year | Age group (years) | Population size ^a^ | Severe acute respiratory infection | | Annual percentage of influenza positive respiratory samples ^d^ | Influenza-associated hospitalizations ^e^ | | Influenza-associated deaths ^f^ | |
| --- | --- | --- | --- | --- | --- | --- | --- | --- | --- |
|  |  |  | Hospitalizations ^b^ | Mortality ^c^ |  | Number  (95% CI) | Rate (95% CI) | Number  (95% CI) | Rate (95% CI) |
|  | <5 | 1075674 | 5734 | 64 | 24/188 (13) | 904 (844 - 963) | 84 (78.5 - 89.5) | 8 (2 - 14) | 0.7 (0.2 - 1.3) |
| 2009 | 5-64 | 6480182 | 1555 | 67 | 216/539 (40) | 560 (512 - 603) | 8.6 (7.9 - 9.3) | 26 (13 - 32) | 0.4 (0.2 - 0.5) |
|  | ≥65 | 320806 | 563 | 62 | 5/12 (42) | 68 (52 - 84) | 21.1 (16.1 - 26.2) | 7 (2 - 13) | 2.2 (0.6 - 3.9) |
|  | <5 | 1079289 | 9203 | 103 | 25/250 (10) | 1304 (1233 - 1374) | 120.8 (114.2 - 127.3) | 15 (6 - 22) | 1.3 (0.6 - 2) |
| 2010 | 5-64 | 6634147 | 1504 | 45 | 111/320 (35) | 439 (391 - 478) | 6.6 (5.9 - 7.2) | 12 (0 - 13) | 0.1 (0 - 0.2) |
|  | ≥65 | 332554 | 596 | 79 | 5/17 (29) | 108 (87 - 128) | 32.3 (26.2 - 38.4) | 15 (7 - 22) | 4.5 (2.2 - 6.7) |
|  | <5 | 1082143 | 6727 | 90 | 34/588 (6) | 321 (285 - 355) | 29.6 (26.3 - 32.8) | 4 (0 - 8) | 0.3 (0 - 0.7) |
| 2011 | 5-64 | 6788047 | 1537 | 59 | 29/159 (18) | 281 (244 - 312) | 4.1 (3.6 - 4.6) | 8 (0 - 7) | 0.1 (0 - 0.1) |
|  | ≥65 | 345123 | 644 | 71 | 3/27 (11) | 54 (39 - 68) | 15.5 (11.4 - 19.7) | 7 (2 - 12) | 2 (0.5 - 3.6) |
|  | <5 | 1085293 | 8901 | 90 | 45/645 (7) | 459 (417 - 500) | 42.2 (38.4 - 46.1) | 6 (1 - 10) | 0.5 (0.1 - 0.9) |
| 2012 | 5-64 | 6941226 | 1448 | 58 | 23/189 (12) | 161 (132 - 180) | 2.3 (1.9 - 2.6) | 7 (0 - 7) | 0 (0 - 0.1) |
|  | ≥65 | 358553 | 698 | 83 | 20/63 (32) | 57 (42 - 72) | 15.9 (11.8 - 20.1) | 8 (2 - 13) | 2.1 (0.6 - 3.6) |

^a^ Honduras census projections

^b^ Number of persons hospitalized during 2009–2012 with severe acute respiratory infection (SARI) proxy diagnoses (ICD-10 codes J9-18)

^c^ Number of deaths among persons hospitalized during 2009–2012 with SARI proxy diagnoses (ICD-10 codes J9-18)

^d^ Number of nasal and pharyngeal specimens annually positive for influenza through immunofluorescence and polymerase chain reaction over total number tested (percentage)

^e^ Estimated by the product of the proportion of samples testing positive for influenza and the number of persons hospitalized with SARI by age-group and month (95% confidence interval). Rate per 100,000 habitants

^f^ Estimated by the product of the proportion of samples testing positive for influenza and the number of persons dying by age-group and month (95% confidence interval). Rate per 100,000 habitants

**Table 5: Influenza-associated hospitalizations and deaths in Nicaragua during 2009–2012**

| Year | Age group (years) | Population size ^a^ | Severe acute respiratory infection | | Annual percentage of influenza positive respiratory samples ^d^ | Influenza-associated hospitalizations ^e^ | | Influenza-associated deaths ^f^ | |
| --- | --- | --- | --- | --- | --- | --- | --- | --- | --- |
|  |  |  | Hospitalizations ^b^ | Mortality ^c^ |  | Number  (95% CI) | Rate (95% CI) | Number  (95% CI) | Rate (95% CI) |
|  | <5 | 680135 | 16665 | 172 | 255/1325 (19) | 1883 (1798 - 1968) | 276.8 (264.3 - 289.3) | 21 (12 - 30) | 3.1 (1.8 - 4.4) |
| 2009 | 5-64 | 4810015 | 4460 | 139 | 1163/2870 (41) | 989 (924 - 1049) | 20.5 (19.2 - 21.8) | 26 (14 - 34) | 0.5 (0.3 - 0.7) |
|  | ≥65 | 252161 | 1456 | 124 | 79/347 (23) | 146 (122 - 170) | 57.9 (48.5 - 67.3) | 10 (4 - 16) | 4 (1.5 - 6.5) |
|  | <5 | 682393 | 21700 | 181 | 108/987 (11) | 1610 (1531 - 1689) | 235.9 (224.4 - 247.5) | 18 (9 - 26) | 2.5 (1.3 - 3.8) |
| 2010 | 5-64 | 4873575 | 4421 | 119 | 157/816 (19) | 645 (595 - 692) | 13.2 (12.2 - 14.2) | 20 (10 - 24) | 0.4 (0.2 - 0.5) |
|  | ≥65 | 259572 | 1927 | 165 | 47/285 (16) | 234 (204 - 264) | 90.3 (78.7 - 101.8) | 20 (11 - 29) | 7.7 (4.3 - 11.1) |
|  | <5 | 683164 | 17848 | 207 | 179/1588 (11) | 857 (799 - 914) | 125.4 (117 - 133.8) | 10 (3 - 16) | 1.5 (0.5 - 2.4) |
| 2011 | 5-64 | 4940295 | 4010 | 127 | 488/1244 (39) | 617 (563 - 662) | 12.4 (11.4 - 13.4) | 17 (5 - 25) | 0.3 (0.1 - 0.5) |
|  | ≥65 | 265487 | 1476 | 110 | 31/276 (11) | 59 (44 - 74) | 22.2 (16.5 - 27.9) | 5 (1 - 9) | 1.8 (0.2 - 3.5) |
|  | <5 | 683426 | 19231 | 190 | 28/648 (4) | 1091 (1027 - 1156) | 159.6 (150.2 - 169.1) | 10 (3 - 16) | 1.5 (0.5 - 2.4) |
| 2012 | 5-64 | 5009233 | 3772 | 135 | 28/304 (9) | 385 (346 - 421) | 7.6 (6.9 - 8.4) | 14 (5 - 20) | 0.2 (0.1 - 0.4) |
|  | ≥65 | 270123 | 1627 | 125 | 18/160 (11) | 120 (98 - 141) | 44.4 (36.4 - 52.3) | 11 (4 - 18) | 4.1 (1.6 - 6.5) |

^a^ Nicaragua census projections

^b^ Number of persons hospitalized during 2009–2012 with severe acute respiratory infection (SARI) proxy diagnoses (ICD-10 codes J9-18)

^c^ Number of deaths among persons hospitalized during 2009–2012 with SARI proxy diagnoses (ICD-10 codes J9-18)

^d^ Number of nasal and pharyngeal specimens annually positive for influenza through immunofluorescence and polymerase chain reaction over total number tested (percentage)

^e^ Estimated by the product of the proportion of samples testing positive for influenza and the number of persons hospitalized with SARI by age-group and month (95% confidence interval). Rate per 100,000 habitants

^f^ Estimated by the product of the proportion of samples testing positive for influenza and the number of persons dying by age-group and month (95% confidence interval). Rate per 100,000 habitants

**Table 6: Sensitivity analysis of influenza-associated hospitalizations and deaths**

| Sensitivity Analysis | Age group (years) | Every inhabitant at risk ^c^ | Public hospital coverage ^d^ | Pandemic period  (2009–2010) ^e^ | Seasonal period  (2011–2012) ^f^ |
| --- | --- | --- | --- | --- | --- |
| Influenza-associated hospitalizations ^a^ Rate (95% CI) | <5 | 113 (91, 134) | 163 (131, 194) | 151 (110, 192) | 75 (54, 95) |
|  | 5-64 | 9 (7, 11) | 13 (10, 16) | 13 (10, 16) | 6 (4, 7) |
|  | >64 | 44 (35, 53) | 61 (49, 73) | 56 (40, 71) | 33 (23, 43) |
| Influenza-associated deaths ^b^ Rate (95% CI) | <5 | 1.3 (0.9, 1.7) | 1.9 (1.4, 2.5) | 1.7 (1.1, 2.2) | 1.0 (0.6, 1.5) |
|  | 5-64 | 0.5 (0.4, 0.6) | 0.7 (0.5, 0.8) | 0.7 (0.5, 0.9) | 0.3 (0.2, 0.4) |
|  | >64 | 7.3 (5.5, 9.1) | 9.9 (7.5, 12.2) | 9.4 (6.1, 12.6) | 5.7 (3.7, 7.7) |

^a^ Estimated by the product of the proportion of samples testing positive for influenza and the number of persons hospitalized with SARI by age-group and month (95% confidence interval). Rate per 100,000 habitants

^b^ Estimated by the product of the proportion of samples testing positive for influenza and the number of persons dying by age-group and month (95% confidence interval). Rate per 100,000 habitants

^c^ We assumed that every inhabitant was at risk of developing severe influenza illness. We estimated the rates using information from all period (2009–2012)

^d^ We assumed that only the proportion of the population typically seeking care at public hospitals network was at risk of developing severe influenza (see methods)

^e^ We estimated the rates only for the pandemic period (2009–2010) assuming that every inhabitant was at risk of developing severe influenza illness

^f^ We estimated the rates only for the seasonal period (2011–2012) assuming that every inhabitant was at risk of developing severe influenza illness
